# Self-Reported Obsession Toward COVID-19 Preventive Measures Among Undergraduate Medical Students During the Early Phase of Pandemic in Jordan

**DOI:** 10.3389/fpubh.2021.719668

**Published:** 2021-11-08

**Authors:** Tariq N. Al-Shatanawi, Samir A. Sakka, Khalid A. Kheirallah, Abdel-Hameed Al-Mistarehi, Shawkat Al-Tamimi, Nasr Alrabadi, Jomana Alsulaiman, Ali Al Khader, Farah Abdallah, Loai Issa Tawalbeh, Tareq Saleh, Waleed Hijazi, Ayham R. Alnsour, Nidal A. Younes

**Affiliations:** ^1^Department of Public Health and Community Medicine, Faculty of Medicine, Al-Balqa Applied University, Al-Salt, Jordan; ^2^Department of Special Surgery, Faculty of Medicine, Al-Balqa Applied University, Al-Salt, Jordan; ^3^Department of Public Health and Family Medicine, Faculty of Medicine, Jordan University of Science and Technology, Irbid, Jordan; ^4^Department of Pharmacology, Faculty of Medicine, Jordan University of Science and Technology, Irbid, Jordan; ^5^Department of Pediatrics, Faculty of Medicine, Yarmouk University, Irbid, Jordan; ^6^Department of Pathology and Forensic Medicine, Faculty of Medicine, Al-Balqa Applied University, Al-Salt, Jordan; ^7^Department of Mental Health, Faculty of Nursing, The Hashemite University, Zarqa, Jordan; ^8^Faculty of Nursing, Al-Bayt University, Al-Mafraq, Jordan; ^9^Department of Basic Medical Sciences, Faculty of Medicine, The Hashemite University, Zarqa, Jordan; ^10^Faculty of Medicine, Al-Balqa Applied University, Al-Salt, Jordan; ^11^Department of Surgery, Faculty of Medicine, Al-Balqa Applied University, Al-Salt, Jordan

**Keywords:** COVID-19, obsession, medical students, knowledge, risk perception, preventive measures, Jordan

## Abstract

**Background:** Coronavirus disease 2019 (COVID-19) pandemic and its associated precautionary measures have substantial impacts not only on the medical, economic, and social context but also on psychological health. This study aimed to assess the obsession toward COVID-19 preventive measures among undergraduate medical students during the early phase of the pandemic in Jordan.

**Methods:** Online questionnaires were distributed between March 16, 2020 and March 19, 2020. Socio-demographic characteristics were collected, and self-reported obsession toward COVID-19 preventive measures was assessed using a single question.COVID-19 knowledge, risk perception, and precautionary measures were evaluated using scales. Using the chi-square test, Student *t*-test, and one-way ANOVA, we assessed the differences in the obsession of students with socio-demographic characteristics and scores of the scales.

**Results:** A total of 1,404 participants (60% were female participants) completed the survey with a participation rate of 15.6%. Obsession with preventive measures was reported by 6.8%. Obsession was significantly more common among women (9.2%) than men (3.3%) and students who attended COVID-19 lectures (9.5%) than those who did not attend such lectures (5.8%) (*p* < 0.001 and *p* = 0.015, respectively). Obsessed participants reported significantly higher levels of COVID-19 knowledge (*p* = 0.012) and precautionary measures (*p* < 0.001). COVID-19 risk perception had a mild effect size difference but with no statistical significance (*p* = 0.075). There were no significant differences in the academic levels of participants (*p* = 0.791) and universities (*p* = 0.807) between students who were obsessed and those who were not.

**Conclusions:** Obsession is one of the significant but unspoken psychological effects of COVID-19 precautionary measures among undergraduate medical students. Medical schools should be equipped with means to handle pandemic psychological effects.

## Introduction

In December 2019, the first Coronavirus disease 2019 (COVID-19) case was reported in Wuhan, China. Subsequently, on March 11, 2020, the COVID-19 spread worldwide and had been classified as a pandemic by the World Health Organization (WHO) (1, 2). In order to limit the spread of the severe acute respiratory syndrome coronavirus 2 (SARS-CoV-2) that caused the COVID-19 pandemic, many countries around the world went into lockdown, including Jordan where the National Defense Law had been operated since March 17. These strict outbreak response measures with massive lockdown applied at early stages contributed to suppressing the infectivity rates of this outbreak initially in Jordan and reducing its impact on public health (3–5). Pandemics are known to have an impact and burden not only on the medical, economic, and social context but also on psychological health (3, 6–8). COVID-19 pandemic and its associated precautionary measures could exacerbate psychiatric symptoms, including stress, anxiety, and fear of being contaminated by germs and dirt, which may lead to disinfecting or washing hands repeatedly until the skin is harmed.

According to the American Psychiatric Association, obsessive–compulsive disorder (OCD) is a chronic psychiatric disease characterized by obsessions that are recurrent, intrusive, unwanted, time-consuming, and distressing thoughts, images, or urges that are impairing and anxiety-increasing. Thus, patients attempt to relieve this anxiety by performing compulsions, which are repetitive behaviors or mental acts. Previous studies indicated that OCD has a lifetime prevalence of 2–3% and could affect up to 3.3% of the general population (9–12). Family history, traumatic, and stressful events like the COVID-19 pandemic and its associated measures, and other mental health illnesses such as anxiety, depression, and substance abuse are risk factors for developing OCD (10, 13, 14). Moreover, obsession behavior has become more apparent and profound during illnesses and infection outbreaks (15–17).

During the COVID-19 pandemic and its preventive measures, the general population, including frontline healthcare workers and medical students, became vulnerable to emotional distress and psychological challenges, including stress symptoms, anxiety, frustration, depression, panic disorder, and fear of SARS-CoV-2 infection, contact with contaminated surfaces, socioeconomic effects of the pandemic, and foreigners (18–23). These psychological problems with the enormous preoccupations of the general population, exposure to often scary news through the media, and the extensive health recommendations by authorities might trigger the obsession about contamination and the possibility of contacting SARS-CoV-2 infection; thereby, stimulating compulsive behaviors such as spending hours disinfecting or washing hands, taking excessively long showers, and not rarely, harming their skin as well as continuously cleaning the surfaces other people have touched, and increased avoidance of others (9, 15, 24–26). Avoidance of situations that could be considered as presenting a high risk of contamination can also occur, such as using public transportation, sitting on a public park bench, or going to a public bathroom (9, 26, 27).

Several studies investigated the potential effects of COVID-19 on obsession behavior and their possible causalities among the general population, including children and adults (14, 24, 25, 28–32). A Canadian study was conducted at an early stage of the COVID-19 pandemic to investigate the prevalence of OCD symptoms among the general population using the brief obsessive-compulsive scale (BOCS) scale (14). The authors found that 60% of the participants had a new onset of OCD symptoms, and more than half engaged in compulsive hand washing during the COVID-19 pandemic at rates significantly higher than prepandemic rates (14). In a crosssectional survey from Saudi Arabia, a neighboring country to Jordan, conducted in July 2020, which included 2,909 respondents from the general population, 58% reported new-onset obsessions, 46% compulsions, and 72% moderate to high perceived stress (32). Germs- and virus-related obsessions and perceived stress were significantly higher among students, quarantine discipliners, and those who spent more days in quarantine (32).

Previous studies reported that psychological distress was more prevalent among frontline healthcare workers than the general population during stressful situations like the COVID-19 pandemic (23, 33–38). A recent systematic review and meta-analysis pooled and analyzed data from 20 studies comprising 10,886 healthcare workers and revealed high prevalence rates of depression (24%), anxiety (29%), insomnia (44%), posttraumatic stress symptoms (26%), phobia (35%), obsessive–compulsive symptoms (16%), and somatization symptoms (11%) among healthcare workers during the COVID-19 pandemic (33). A cross-sectional study involving a total of 198 participants in Turkey reported that healthcare workers in the COVID-19 section had significantly increased obsessive–compulsive disorders, depression, and anxiety (34). In comparison, an extensive survey from frontline health care workers in China documented that the prevalence rates of psychological disorders, including depression, anxiety, somatization symptoms, and insomnia, among frontline medical staff were significantly higher than those in the general population (38).

Obsessive–compulsive disorder develops mainly during adolescence and late teens, with a mean age of 19–20 years for OCD onset (39, 40). Thus, university students are vulnerable to develop OCD symptoms. Moreover, previous studies reported that the prevalence rate of OCD among university students is double that of the general population. Furthermore, the young age group is more prone to mental hazards, including suicidal attempts and substance use, which are associated comorbidities for OCD (40–42).

As undergraduate medical students are closely associated with health care workers, they are susceptible to experience similar psychological and emotional distress. Also, their dense curriculum, limited leisure time, the stressful nature of medical schools, and as the medical students are asked to be more precise, perfect, and obsessive a little bit more, they are at high risk for developing OCD (43–45).

COVID-19 pandemic and its precautionary measures had substantially adverse effects on the undergraduate medical students as their clinical training was almost blocked, rotations were altered or canceled to maximize the capacity of healthcare systems for COVID-19 cases. As well, the closure of medical schools, clinical training disturbance, and the laboratories and classroom lectures shifted toward distant online lessons, leaving them to continue their studies remotely (46–49). As a result, they faced challenging circumstances with continuing their studies, making them even more vulnerable to psychological disorders. In addition, the increasing efforts of handwashing and general hygiene as an essential step in COVID-19 prevention might trigger the obsession with contamination and compulsive hand washing, which are reported as common symptoms of OCD (25, 50). Previous reports on the undergraduate medical students at Jordanian universities indicated high levels of adopting anti-COVID-19 precautionary measures, including social isolation strategies, avoiding crowded places, canceling traveling plans, canceling social events, changing life habits, regular hand washing, and enhanced personal hygiene measures (51, 52). However, these studies did not measure the potential psychological effects of these preventive measures among such a vulnerable group of the population.

Obsessive–compulsive disorder symptoms could adversely affect the general well-being, academic performance, and social interactions of students. Such issues might have a considerable impact on the quality of life unless being detected early and properly managed (53, 54). In the face of this stressful situation, and to limit the damage effects of the COVID-19 pandemic and future pandemics and their associated precautionary measures on the future frontline healthcare workers, it is essential to figure out the extent of psychological impact and learning difficulties they are experiencing. This may help formulate policies and strategies to support the well-being of the medical students and break this vicious cycle of stress and learning difficulties through adaptive flexibility for curriculum innovation, culturally sensitive resilience, and well-being interventions.

Several studies investigated the psychological impact of the COVID-19 pandemic on undergraduate medical students, including stress, anxiety, depression, and sleep problems. However, data regarding the assessment of obsession toward COVID-19 preventive measures in such vulnerable groups is scarce. Therefore, in this study, we shed light on the psychological obsession as extreme mental effects of COVID-19 preventive measures on medical students at the undergraduate level in light of the multiple other reports that studied the general psychological and mental effects of the COVID-19 pandemic on the general population, health professionals, and medical students as a general stressor on health and communities (20–22, 33). Thus, this exploratory study aimed to estimate the prevalence rates of self-reported obsession toward COVID-19 preventive measures among undergraduate medical students in Jordan during the early phase of the COVID-19 pandemic. Moreover, the potential differences in the reported obsession of students and its determinants were assessed in the light of socio-demographic characteristics, COVID-19 knowledge, risk perception of COVID-19 susceptibility, and commitment levels to COVID-19 precautionary measures.

## Materials and Methods

### Study Design and Ethical Considerations

An anonymous, cross-sectional, web-based, exploratory survey was conducted online. Participants were eligible if they were living in Jordan, aged 18 years and above, undergraduate, and attending one of the medical schools in Jordan. Thus, the questionnaire included questions about the living area, age, whether enrolled in Jordanian medical school, academic year (first to the sixth year), and university name to ensure that participants met the inclusion criteria. The data was collected during the entire lockdown period between the 16th and 19th of March, 2020. The research team members developed the survey through the Google Form tool and posted it on the online platforms of all Jordanian medical schools. Participants did not receive any compensation or rewards for their participation in the study.

All procedures performed in this study were approved by the Institutional Review Board (IRB) committees at Al-Balqa Applied University and Hashemite University. This study was conducted following the 1975 Helsinki declaration, as revised in 2008 and its later amendments or comparable ethical standards. An electronic informed consent form was available and signed by all the participants at the beginning of the online survey and includes detailed information about the purpose, objectives, procedure, and IRB approval of the study. Moreover, students were informed that their participation was voluntary, and they could terminate the survey at any time desired. The data was kept confidential, as all information was de-identified, and identifier-related questions such as participant name, the university identified number, and place of residence were not asked. Also, a study-specific unique number was created for each participant, and this file was locked and password-protected with limited access and authorizations only to the research team to view, share, and use it. All analyses and further works were performed on this deidentified file. This study was part of a more extensive study conducted to assess knowledge, attitudes, and precautionary measures toward COVID-19 among medical students (51).

### Population and Sampling Procedure

Jordan is a small developing country located in the Middle East and North Africa region with a population size of ~11 million. Jordan has six medical schools throughout the country, including Jordan University, Jordan University of Science and Technology, Hashemite University, Al-Yarmouk University, Al-Balqa' Applied University, and Mu'tah University. The Doctor of Medicine curriculum in Jordanian universities is a 6-year undergraduate degree. During the first 3 years, the basic medical and behavioral sciences are taught to the undergraduate medical students, the so-called preclinical level. The clinical skills and rotations are provided within the latter 3 years; thereby, called clinical level. The country has been affected by the COVID-19 pandemic since March 2, 2020 (55, 56). The primary research aim of this study was to assess the prevalence estimates of self-reported, the obsession of COVID-19 precautionary measures among undergraduate medical students in Jordan. According to the Jordanian Ministry of Higher Education and Scientific Research, the total number of undergraduate medical students enrolled in Jordanian medical schools was ~9,000 at the time of conducting this study. Thus, with an estimated obsession prevalence rate of 50% as the most conservative assumption and a margin of error of 4% [99% confidence interval (CI): 46–54%], we calculated a sample size of 933 individuals. To increase the power of our study, we aimed to include over 1,000 undergraduate medical students from all Jordanian universities and all years of enrollment (from the first year to the sixth year).

### Survey Instruments

The online questionnaire was designed based on the frameworks of previous studies to assess the COVID-19 knowledge, the risk perception of COVID-19 susceptibility, and the conducted precautionary measures toward COVID-19 (57–65). Regarding obsession measurement, previous studies investigated the prevalence estimates of OCD, its severity, exacerbation, and correlates during the COVID-19 pandemic using several validated scales (14, 25, 31, 32, 50, 66). However, the novelty of our study was in its objectives as it assessed the obsession toward COVID-19 preventive measures precisely, which could not be measured using the previous measuring tools for OCD. Thus, due to the lack of validated tools for measuring obsession toward COVID-19 preventive measures in the Jordanian population, the authors incorporated a new single question, in the light of previously used scales, to evaluate the self-reported obsession of participants toward COVID-19 preventive measures.

The questionnaire was first developed in English and then translated to Arabic, the official language of Jordan. It was then translated back into English by two independent translators and compared by a third one. Validity was checked in a pilot study that included 20 random undergraduate medical students who assessed the clarity of the questionnaire, and no significant modifications were required. The survey tool used in this study consisted of four parts.

The first part of the questionnaire included questions about socio-demographical details and characteristics of the participants, including living area, gender, academic year, name of the enrolled university, and the attendance of lectures about COVID-19. It also included a question that assessed the self-reported obsession with COVID-19. The latter question was close-ended with five options, including in order “I am concerned and cautious,” “I changed daily preventive behaviors,” “I am concerned but not cautious,” “I do not care at all,” and “I become obsessed by preventive measures.” Thus, the participant who answered the last option was considered a case with a high risk of obsession toward COVID-19 preventive measures.

The second part of the survey included a scale of 11 items to assess the knowledge of students about COVID-19. The scale assessed how much the participants knew about COVID-19 using multiple factual questions about the nature of the disease and facts about preventive strategies. The questions were extracted from the latest WHO report and U.S. Centers for Disease Control and Prevention (CDC) guidelines on March 12, 2020. The students were asked 11 questions, including the disease etiology (virus, bacteria, fungus, or others), “Is COVID-19 a respiratory infection? with Yes or No response,” “Is coronavirus contagious? Yes or No,” the ways of COVID-19 transmission (airborne, droplets, touching, or I do not know), coronavirus viability on surfaces (minutes to hours, hours to days, or I do not know), counting ≥ three signs and symptoms of COVID-19 (fever, cough, shortness of breath, chest pain, rhinorrhea, etc.), high-risk group (elderly, diabetics, having cardiovascular diseases, having chronic respiratory disease, immunocompromised, having cancer, or I do not know), the incubation period of the disease (1–14, 15–30, or >30 days), the worldwide mortality rate of the COVID-19 confirmed cases (<0.5, 0.5–10, 10–30, >30%), availability of vaccines (Yes or No), and counting ≥ three COVID-19 prevention strategies (facial mask use, social distancing, avoidance of crowded places, hands washing, use of disinfectants, etc.). Each item answer was scored one for the correct answer and zero for the wrong answer. The knowledge scale scores of items were summed for each participant and ranged from 0 to 11, with a higher score indicating higher knowledge about COVID-19. The Cronbach's alfa (α) for items on the knowledge scale was 0.663.

The third part of the questionnaire included the risk perception of the COVID-19 susceptibility scale with six items and a 5-point Likert scale for each item. This scale was previously conducted on international samples of 6,991 participants from 10 countries across Europe, North America, Australia, and Asia (63). Also, it was designed following previous studies on risk perception (64, 65, 67, 68). The first question was “How worried are you personally about Coronavirus/COVID-19 at present?” with answer options and scores: “0 = not at all worried/not worried/neutral,” whereas “1 = worried/very worried.” The second and third items assessed the perceived likelihood of a person catching the virus, and their family and friends over the next 6 months. The answers were collapsed into “0 = Not at all worried/not worried/neutral, and 1 = very worried/worried.” The fourth item was “How much do you agree or disagree with the coronavirus will NOT affect very many people in the country I am currently living in.” The 5-point Likert code was reversed in this question to be “0 = strongly agree/agree/neutral and 1 = strongly disagree/disagree.” The last two items were “How much do you agree or disagree with, I will probably get sick with the coronavirus” and “How much do you agree or disagree with getting sick with the coronavirus can be serious.” The answers of the last two items were collapsed into “0 = strongly disagree/disagree/neutral, and 1 = strongly agree/agree.” The sum of the scores of these six items was calculated for each participant, and the total score was reported. The Cronbach's α of the scale items was 0.732. A higher score indicates a higher risk perception of COVID-19 susceptibility among the participants.

The fourth part included a 21-item scale to assess the adaption of precautionary measures toward COVID-19 (51, 58, 61). Different precautionary measures were assessed by asking the students, “How often have you engaged in the following behaviors?” These behaviors included: buying a face mask, wearing a face mask, washing hands regularly, use disinfectants regularly, buying, and using a “Portable Air Doctor,” which is a portable product that helps avoid viruses, bacteria, and fungi to come in contact with the user within a 1-m radius, paying more attention to personal hygiene, staying at home as much as possible, avoiding contact with specific groups of the population, avoiding public gatherings, paying attention to a balanced diet, cleaning or disinfecting my phone regularly, avoiding eating outside, avoiding using public transportation, avoiding handshaking when greeting others, avoiding kissing of others when greeting them, getting sufficient sleep, closely monitoring personal physical health, closely monitoring the physical health of the people around you, following social distancing, increasing fluid intake, and persuading people around to follow precautionary guidance. Each item of the COVID-19 precautionary measures scale has five Likert points, including “All the times, Often, Sometimes, Rarely, or Never.” In the analysis phase of collected data, the five points were collapsed into three scores: “all the times or often” and was given the score of two, “sometimes or rarely” and was given the score of 1, and “Never” and was given the score of 0. This approach of collapsing was validated to be used (69). The Cronbach's α for these items was 0.882. The scores of the items were summed to provide a total score for each participant, ranged from 0 to 42, with a higher score indicating a higher commitment level to COVID-19 precautionary measures.

### Statistical Analysis

The data were analyzed using the Statistical Package for Social Science (SPSS) version 23. Descriptive statistics (frequencies and percentages) were calculated for the sample socio-demographic characteristics and the self-reported obsession. Continuous variables, including scores of scales, were presented as mean ± standard deviation (m ± SD) after verifying the normality of the dataset. Internal consistency reliability was measured using Cronbach's α for each of the three scales. Several assumptions underlying Cronbach's α that were tested and met each used scale, including the items of the scales were ordinal, and the scales were unidimensional. We assessed the correlations between the error terms in the regression models of each item and the total scale score, and there were no significant correlations between error terms. More importantly, the assumption that the items were tau-equivalent was also met as revealed by the factor analysis test (70–72).

The univariate analyses were conducted to assess the differences in the socio-demographic characteristics of participants (gender, academic level, enrolled university, and attending lectures about COVID-19), and the scores of COVID-19 knowledge, risk perception of COVID-19 susceptibility, and precautionary measures toward COVID-19 between those who reported obsession by COVID-19 preventive measures and those who did not report such a complaint. The univariate analyses were completed using a chi-square test for categorical variables and Student's *t*-test or one-way ANOVA for continuous variables.

Avoidance of the possibility of assumptions violation was checked when analyzing data using Student's *t*-test or one-way ANOVA (73). A Shapiro–Wilk test showed that the data were approximately normally distributed with acceptable *z*-scores for the skewness and keratosis values located within the range of −1.96 and +1.96 (74–76). Also, based on the central limit theorem, our sample size is sufficiently large enough to conduct parametric statistical tests such as Student's *t*-test and one-way ANOVA (77). Box plots were conducted to ensure that the data was free from outliers, which can be identified as those points that lie beyond the whiskers of the plot. Homogeneity of variance was assessed using Levene's test for equality of variances. To meet the assumption of homogeneity of variance, the *p*-value for Levene's test should be above 0.05 (78). All the samples were drawn independently of each other, and within each sample, the observations were sampled randomly and independently of each other.

A binary logistic regression analysis was used to determine the risk factors of developing obsession toward COVID-19 preventive measures. This approach is an efficient and powerful way to measure the associations, predict the outcomes, and control confounding effects of variables (79, 80). The dependent variable was the self-reported obsession of participants toward COVID-19 preventive measures. Model selection using the stepwise backward approach with a cutoff *p*-value of 0.2 was used to determine the confounding factors (81–83) and select the final parsimonious model where gender, academic level, attending university, attending lectures about COVID-19, COVID-19 knowledge, risk perception of COVID-19 susceptibility, and precautionary measures toward COVID-19 were included as explanatory variables. The variables in the last model were checked for multicollinearity using variance inflation factor (VIF). Odds ratios (OR), 95% confidence intervals (95% CI), and *p-*values were reported. Statistical significance was considered at a *p-*value of ≤ 0.05, whereas practical significance was represented by effect sizes using Cohen's d (standardized mean difference) for continuous variables and OR for categorical variables (84).

## Results

### Characteristics of Participants

**Figure 1:**
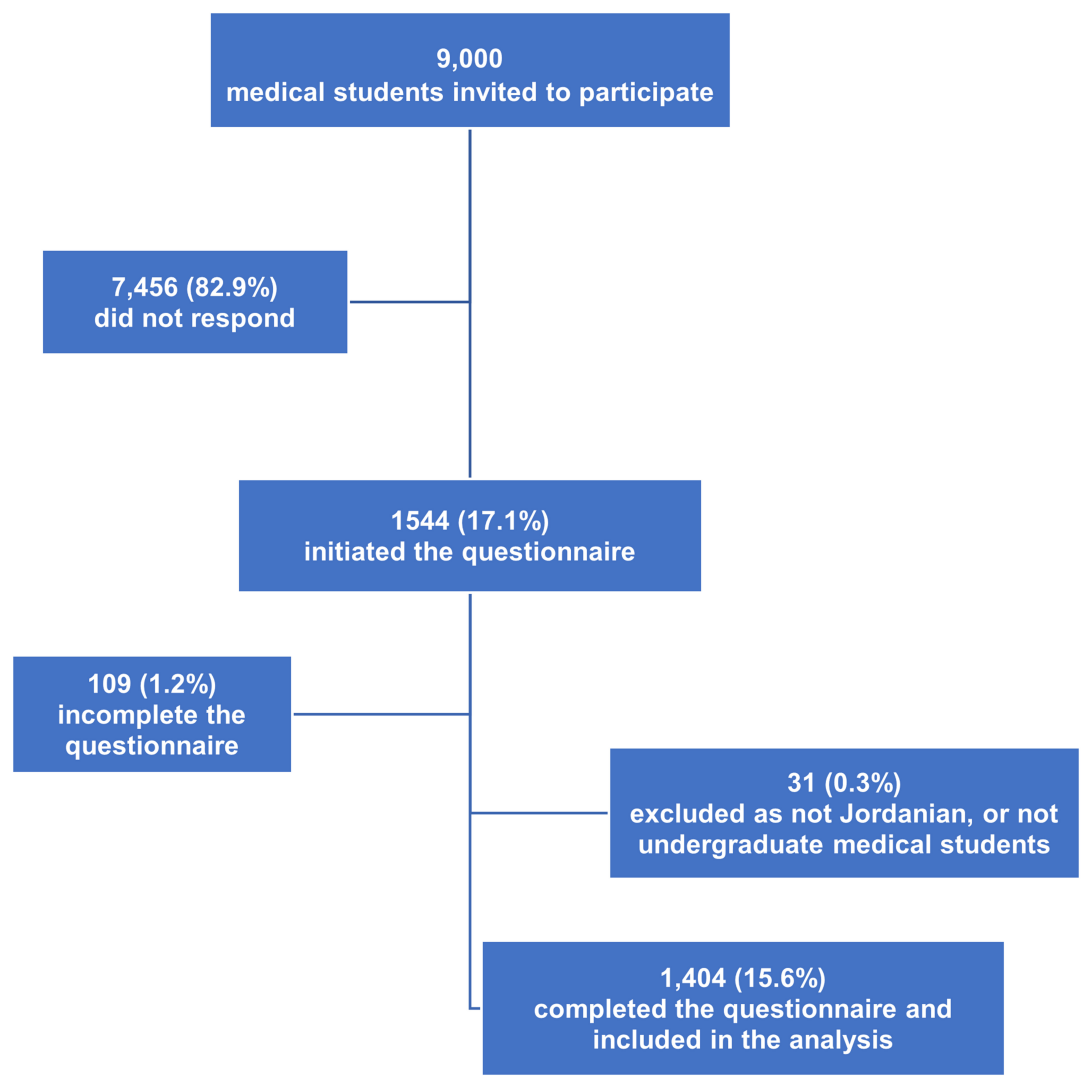
Flow chart of the participants.

A total of 1,404 undergraduate medical students completed the survey and were included in the study analysis with an estimated participation rate of 15.6% (Figure 1). Most of the participants (*n* = 835, 59.5%) were identified as cis-gender women. The sample was collected from six medical schools in Jordan. The most considerable bulk of the participants was from the University of Jordan (*n* = 549, 39.1%) and Jordan University of Science and Technology (*n* = 362, 25.8%). The results also showed that 59.6% (*n* = 837) of the participants were students in their preclinical level, whereas 40.4% (*n* = 563) were in their clinical level. About three-quarters of the participants (72.2%) did not attend any lecture about COVID-19. The socio-demographic characteristics of the participants are presented in Table 1.

**Table 1:** Socio-demographic characteristics of participated medical students (*n* = 1,404).

**Variable**	**Number**	**Percentage**
**Cis-genderism**		
Male participants	569	40.5
Female participants	835	59.5
**University**		
Jordan University (JU)	549	39.1
Jordan University of Science and Technology (JUST)	362	25.8
Hashemite University (HU)	193	13.7
Al-Yarmouk University	122	08.7
Al-Balqa' Applied University	116	08.3
Mu'tah University	62	04.4
**Academic year**		
First year	145	10.3
Second year	349	24.9
Third year	343	24.4
Fourth year	263	18.7
Fifth year	176	12.5
Sixth year	128	09.1
**Attending lectures about COVID-19**		
Yes	391	27.8
No	1,013	72.2

### Self-Reported Obsession, Knowledge, Risk Perception of Susceptibility, and the Level of Precautionary Measures Toward COVID-19

**Figure 2:**
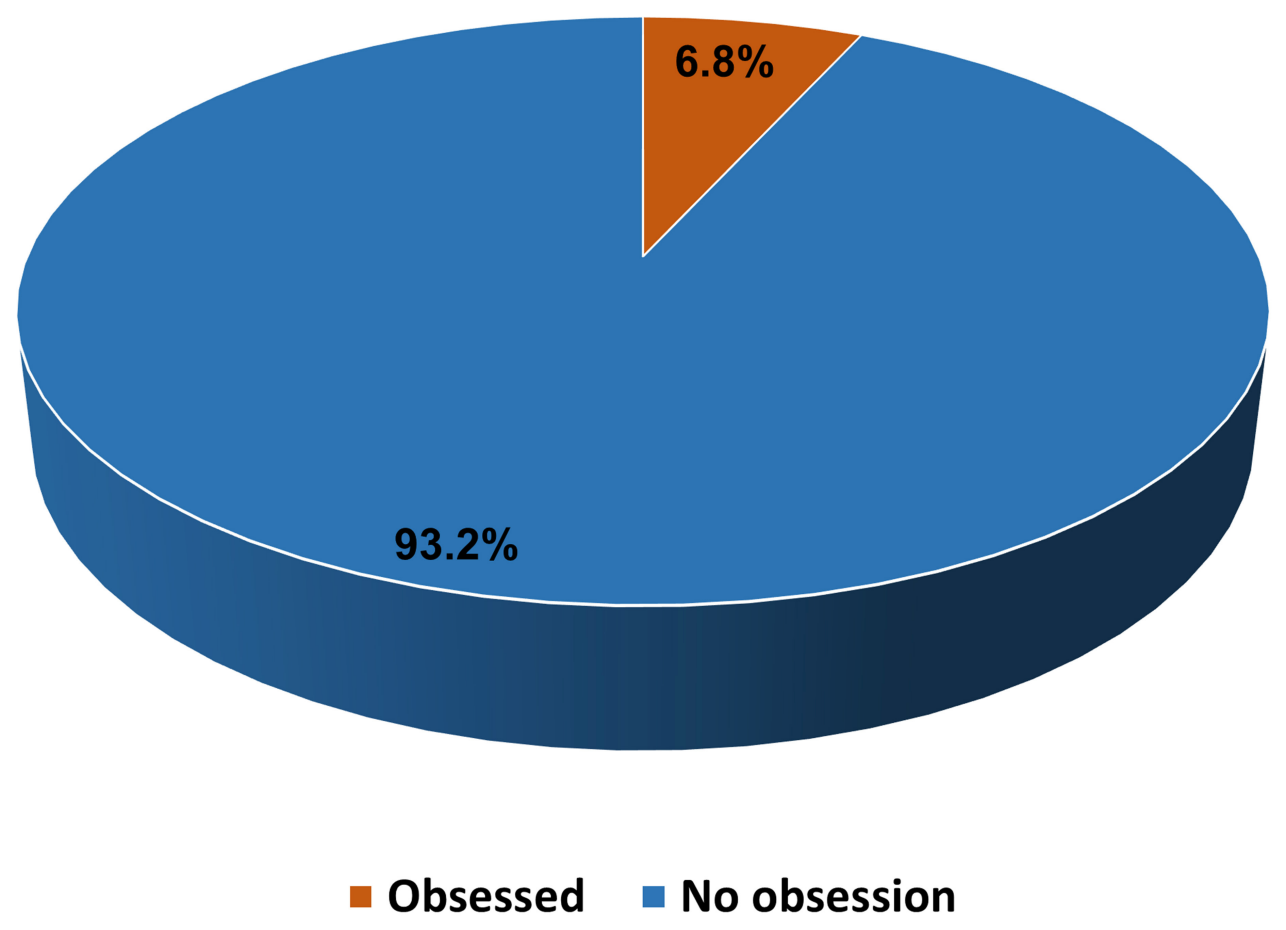
Self-reported obsession toward the COVID-19 preventive measures among undergraduate medical students.

The results showed that 6.8% (*n* = 96) of the participants reported obsession by preventive measures toward COVID-19, whereas 93.2% (*n* = 1,308) were not obsessed, but they were concerned and cautions (45.5%), changed daily preventives measures (31.5%), concerned but not cautious (13.1%), and the least percentage of participants (3.1%) did not care at all (Figure 2).

The mean score of knowledge about COVID-19 among medical students was 6.68 (SD = 2.21) and ranged between 1 and 11. On the other hand, the mean risk perception score of COVID-19 susceptibility was 2.66 (SD = 1.25) and ranged between 0 and 6. The mean score of the COVID-19 precautionary measures scale among the participants was 29.44 (SD = 7.01) and ranged from 0 to 42 (Table 2).

**Table 2:** The levels of knowledge, risk perception, and precautionary measures of COVID-19 among medical students.

**Variable**	**Number of items**	**M (SD)**	**Actual range**	**Possible range**
Knowledge about COVID-19	11	6.68 (2.21)	1–11	0–11
Risk perception of COVID-19 susceptibility	6	2.66 (1.52)	0–6	0–6
Precautionary measures of COVID-19	21	29.44 (7.01)	0–42	0–42

*M, Mean; SD, Standard Deviation*.

### The Differences of Self-Reported Obsession by the Socio-Demographic Characteristics, COVID-19 Knowledge, Risk Perception of COVID-19 Susceptibility, and COVID-19 Precautionary Measures of Medical Students

The self-reported obsession toward COVID-19 preventive measures was significantly differed by the socio-demographic characteristics of the enrolled medical students (Table 3). Female medical students were more likely to be obsessed with preventive measures (9.2%) than male students (3.3%), with an unadjusted OR of 2.94 (95% CI 1.76–4.92, *p* < 0.001). Also, the prevalence rates of self-reported obsession toward COVID-19 preventive measures were significantly higher among students who attended lectures about COVID-19 (9.5%) than those who did not attend such lectures (5.8%), with an unadjusted OR of 1.69 (95% CI 1.10–2.59, *p* = 0.015). However, there were no statistically significant differences in the self-reported obsession with both academic level (*p* = 0.791) and the enrolled university (*p* = 0.807). Also, no practical significance was noted as the unadjusted OR was 1.06 and 1.02 for the academic level and the enrolled university, respectively, with self-reported obsession.

**Table 3:** The differences of self-reported, COVID-19 preventive measures related obsession by the socio-demographic characteristics of medical students.

**Socio-demographic and clinical variable**	**Obsession by preventive measures**, ***n*** **(%)**		***p*-value**
	**Yes, *n* = 96 (6.8)**	**No, *n* = 1,308 (93.2)**	
**Cis-genderism**			*<0.001*
Male participants, *n* = 569	19 (3.3)	550 (96.7)	
Female participants, *n* = 835	77 (9.2)	758 (90.8)	
**University**			*0.807*
Jordan University (JU), *n* = 549	36 (6.6)	513 (93.4)	
Jordan University of Science and Technology (JUST), *n* = 362	29 (8.0)	333 (92.0)	
Hashemite University (HU), *n* = 193	12 (6.2)	181 (93.8)	
Al-Yarmouk University, *n* = 122	9 (7.4)	113 (92.6)	
Al-Balqa Applied University, *n* = 116	8 (6.9)	108 (93.1)	
Mu'tah University, *n* = 62	2 (3.2)	60 (96.8)	
**Academic level**			*0.791*
Pre-clinical level, *n* = 567	40 (7.1)	527 (92.9)	
Clinical level, *n* = 837	56 (6.7)	781 (93.3)	
**Attending lectures about COVID-19**			*0.015*
Yes, *n* = 391	37 (9.5)	354 (90.5)	
No, *n* = 1,013	59 (5.8)	954 (94.2)	

*The italic values represent p-values*.

An independent student *t*-test was used to examine the differences in the mean scores of COVID-19 knowledge, risk of susceptibility, and precautionary measures scales between medical students who self-reported obsession toward COVID-19 preventive measures and those who did not (Table 4). A Levene's test verified the equality of variances in the samples (homogeneity of variances) with a *p* > 0.05 for each scale. The students who had high levels of adapted precautionary measures toward COVID-19 (M = 33.414, SD = 5.95.57) were significantly more obsessed with preventive measures than those with low levels of precautionary measures (M = 29.816, SD = 6.10) with a mean difference of −3.6 [*t*_(1,402)_ = −5.6, *p* < 0.001]. This difference was statistically and practically significant with a *p*-value of <0.001 and a moderate effect size (Cohen's *d* = 0.62). Moreover, the results showed that the mean score of the COVID-19 knowledge scale was significantly higher among those who self-reported to be obsessed (M = 7.2, SD = 2.3) compared with their non-obsessed counterparts (M = 6.6, SD = 2.2), with a mean difference of −0.6 [*t*_(1,402)_ = −2.5, *p* = 0.012] and mild effect size (Cohen's *d* = 0.26). However, there was no statistical significance in the risk perception of COVID-19 susceptibility between students obsessed with COVID-19 preventive measures (M = 2.9, SD = 1.6) and those who were not (M = 2.6, SD = 1.5) (*p* = 0.075), but there was a mild practical significance with a Cohen's *d* of 0.20.

**Table 4:** The self-reported obsession by preventive measures of COVID-19 and the COVID-19 knowledge, risk perception, and precautionary measures among medical students.

**Variables**	**Obsessed by preventive measures (M, SD)**	**Not obsessed by preventive measures (M, SD)**	**Unstandardized mean difference**	***t* (1,402)**	***p*-value for difference**	**Levene's test statistics**		**Cohen's d effect size (standardized mean difference)**
						***F***	***p*-value**	
Knowledge about COVID-19	7.23 (2.32)	6.64 (2.21)	*−0.59*	*−2.51*	*0.012*	0.292	*0.589*	–0.26
Risk perception of COVID-19 susceptibility	2.93 (1.62)	2.64 (1.51)	*−0.29*	*−1.78*	*0.075*	0.865	*0.353*	–0.20
Precautionary measures of COVID-19	33.34 (5.53)	29.75 (6.05)	*−3.58*	*−5.63*	*<0.001*	2.525	*0.112*	–0.62

*M, Mean; SD, Standard Deviation. The italic values represent p-values*.

### Factors Associated With the Self-Reported Obsession

Using binary logistic regression analysis, cis-female genderism (OR: 2.73, 95% CI: 1.61–4.62, *p* < 0.001), attending lectures about COVID-19 (OR: 1.57, 95% CI: 1.01–2.43, *p* = 0.047), having higher COVID-19 knowledge scores (OR: 1.12, 95% CI: 1.01–1.23, *p* = 0.027), and higher levels of precautionary measures (OR: 1.10, 95% CI: 1.06–1.15, *p* < 0.001) were independent risk factors for obsession toward COVID-19 preventive measures (Table 5).

**Table 5:** The predictors of self-reported obsession by preventive measures of COVID-19^*^.

	**Adjusted Odds ratio**	**95% Confidence interval**	***p*-value**
Cis-female genderism	2.726	1.609–4.620	*<0.001*
Attending lectures about COVID-19	1.565	1.007–2.433	*0.047*
Knowledge about COVID-19	1.117	1.013–1.231	*0.027*
Risk perception of COVID-19 susceptibility	1.129	0.983–1.296	*0.086*
Precautionary measures of COVID-19	1.101	1.055–1.149	*<0.001*

* *Cis-genderism, academic level, university, attending lectures about COVID-19, and scores of COVID-19 knowledge scale, risk perception of COVID-19 susceptibility scale, and precautionary measures toward COVID-19 scale were included as explanatory variables in the backward stepwise binary logistic regression model. The italic values represent p-values*.

## Discussion

This study is one of the first exploratory surveys that shed light on the COVID-19 preventive measures related obsession among undergraduate medical students in a developing country during the early phase of the pandemic. Around 7% of the undergraduate medical students who participated in our study declared self-reported obsession toward COVID-19 preventive measures. Female participants and those who attended lectures about COVID-19 were more likely to report obsession than their counterparts. Being a woman, having a higher commitment to routine and daily-adapted precautionary measures toward COVID-19, and having higher levels of COVID-19 knowledge were independent significant risk factors for developing obsession toward COVID-19 preventive measures. COVID-19 risk perception had a mild effect size difference between students who were obsessed with COVID-19 preventive measures and those who were not, but with no statistical significance. These results necessitate swift interventions to mitigate the psychological impacts of the COVID-19 pandemic and its precautionary measures on medical students. The need for preparation and intervention to mitigate the challenges of the psychological impact of previous epidemics in general and COVID-19, in particular, has been highlighted by several studies (6, 85–87).

With the pandemic progressing, its effect on obsession behavior emerges from different countries on medical and non-medical personnel, including students (14, 21, 28, 66, 88, 89). At the early stages of the COVID-19 pandemic in Jordan, our study showed that around 7% of medical students reported an obsession with COVID-19 preventive measures, such as hand washing, using disinfectants, and wearing masks, etc. This effect may be partly due to unpredictability and uncertainty about the disease and its risk seriousness (90, 91). Female medical students were significantly more obsessed with preventive measures than male (9.9 vs. 3.3%, respectively). This gender difference is consistent with the findings of previously reported studies in the general population (66, 92, 93).

On the other hand, those students who self-reported to be obsessed with the pandemic preventive measures had significantly higher knowledge about COVID-19. As well, students keen to attend COVID-19 related lectures more frequently reported obsession than their counterparts. This finding could be attributed to the fact that fearful and obsessed individuals are more likely to attend such lectures or, alternatively, that the awareness of the disease and its potential consequences during the lectures unpredictability raises the fear among the attendants and subsequently increasing the levels of obsession toward COVID-19 preventative measures (91). This uncertainty about the cause and effect is similar to the chicken and the egg causality dilemma that could not be resolved easily. However, the obsession of students was not statistically different by the risk perception of COVID-19 susceptibility; thereby, we could not confirm the link between obsession and fear of COVID-19. Furthermore, both the attended universities and the academic levels of the students, which might reflect different background levels of knowledge, have had no effects on obsession levels; therefore, the second alternative scenario could not be confirmed.

Several surveys from different countries studied the impact of the COVID-19 pandemic on the mental health status of undergraduate medical students, including stress, anxiety, depression, and sleep problems (22, 94, 95). However, fewer studies included the assessment of the obsession of students. Ji et al. investigated the possible effects of the COVID-19-fear-invoking environment on obsessive–compulsive symptoms among university students at the early pandemic stages in China in the light of their knowledge and fear about COVID-19 (89). The authors found that 11.3% of participants initially scored as possible candidates for OCD. Subsequently, at later stages of the study, around 3.6% of participants had scores indicative of possible OCD. They concluded that the fear and anxiety of COVID-19 had been associated with a greater OCD indicative score, suggesting that the environment of COVID-19 pandemic interaction with the personal psychology, fear, and anxiety of adverse events might be involved in OCD etiology (89).

Moreover, the increase of obsessive tendency and its possible etiology mechanisms concerning the COVID-19 pandemic was studied by Wheaton et al. (91). They investigated the possible relations between health anxiety symptoms and OCD symptoms with the concerns about the spread of COVID-19 during the early stages of the outbreak in the United States. They revealed that concern about COVID-19 spread was moderately and positively correlated with health anxiety symptoms, OCD, and uncertainty intolerance. Also, they reported that intolerance of uncertainty partially accounted for the connections between concern about COVID-19 spread and OCD and health anxiety symptoms (91). Several previous studies suggested a link between the excessive concern about the COVID-19 pandemic and OCD, health anxiety, and recently emerging data (96–98).

### Strengths and Limitations of the Study

The study timelines at the early phase of the COVID-19 pandemic in Jordan are one of its strengths. Also, the reasonably large sample size and its approach in examining a wide range of socio-demographic factors and beliefs to assess the obsession are other strengths of this study. Most previous studies investigated the prevalence estimates of OCD, its severity, exacerbation, and correlations during the COVID-19 pandemic. Thus, our study is one of the first to shed light on the link between obsession and anti-COVID-19 preventive measures in specific. Also, the novelty of this study is in sampling and targeting undergraduate medical students as a vulnerable group of the population not usually reported in international journals. Most vitally, this study is one of the first ones to be conducted in an Eastern Mediterranean developing country with a middle-income. Thus, in the light of literature dominance by researchers in white, high-educated, industrialized, high-income, and democratic countries, we tried to fill the gap of psychological literature regarding such issues outside Western countries, which would contribute to creating global psychological theories (99–101).

Despite the strengths of this study, several limitations should be reported. First, the online nature of the survey where the possibility of e-survey replication by the same individuals cannot be excluded, the results are subject to recall, and we could not check the accuracy of the responses of the participants. However, the large sample size would minimize these effects. Previous studies have shown that an online-based survey is a cost-effective method that could reach effectively targeted people otherwise unreachable and provide a comfortable, private, and safe environment for the respondents to answer questions honestly and accurately compared with face-to-face interviews (102, 103). We suggest inserting one of the several forms of instructional manipulation check, such as a blue-dot task, to increase the statistical power and reduce the signal-to-noise ratio (104, 105). Second, the reported obsession about the COVID-19 preventive measures was relayed on the self-reporting by the participant using a single closed-end question rather than a validated scale. However, there is no validated tool for measuring obsession toward COVID-19 preventive measures in the Jordanian population. Thus, developing a validated Arabic tool for assessing such obsessions among the Jordanian population is highly recommended. Also, clinical interviews by an expert psychiatrist were not conducted to confirm the diagnosis of obsession. However, the self-reported obsession by participants was significantly associated with the precautionary measures scores of the participants, indicating the suitability of the used question for the aims of this study.

Third, the cross-sectional design of the study and the lack of data on the obsession of participants before the COVID-19 outbreak could affect the interpretation of the results. Thus, we could neither provide evidence for causal associations nor the prevalence trends of obsession before and during the COVID-19 crisis. Fourth, although the relatively large sample size of undergraduate medical students was collected from all medical schools throughout the country, the results were unlikely to be generalizable beyond the people who responded due to lack of information about the non-respondents and the targeted population in Jordan, which might cause low achieved representativeness of the population. Also, the participants needed access to a smartphone/computer, which may cause a selection bias, and the response rate was low (16%). However, data completeness was very high, and the participation rate in this study is concordant with previous internet-based surveys (65, 106, 107). Thus, a snowball sampling recruitment method with appropriate incentives is suggested in future studies. Finally, the percentage of students with obsession was small (around 7%), and there was a considerable difference in sample size between obsessed and non-obsessed groups. However, the overall sample size was large (*n* = 1,404); thereby, a sufficient number of obsessed students were studied and analyzed.

## Conclusion

The COVID-19 pandemic will find its end; however, its effects on the mental health and well-being of undergraduate medical students and health care professionals will have longer-lasting detrimental consequences. This large-scale survey of undergraduate medical students reveals the existence of significant but unspoken obsession as one of the psychological impacts of the COVID-19 preventive measures. Multiple demographics, epidemics, and psychological factors, such as cis-female genderism, attending lectures about COVID-19, having higher levels of COVID-19 knowledge, higher commitment to routine and daily-practiced precautionary measures, and increased worries for the self, family, and friends about contracting the disease, surviving if contracted with COVID-19, and its possible complications were found to be significant predictors of obsession among the undergraduate medical students. OCD toward COVID-19 preventive measures could have unignorable effects on medical students.

Neglecting the psychological aspect of the COVID-19 pandemic and its preventive measures would affect the quality of life of medical students, future frontline healthcare workers, and the overall performance of the healthcare system. Therefore, longitudinal awareness of such effects is crucial as OCD symptoms are often hidden; hence, medical schools are invited to identify and treat this condition as early as possible. Mental health services and support should be provided to those students at high risk of OCD. Also, practical plans, devised strategies, and effective swift interventions to safeguard the mental health of such a vulnerable group of population are recommended. We believe our findings would assist the public health stakeholders and medical educators in capturing, mitigating, and remedying the psychological effects of the COVID-19 pandemic, which could be worse than the current pandemic itself. Further studies to investigate the temporal pattern of changes in the mental health status of medical students and to measure the psychological effects of the pandemic and its preventive measures on other college students are recommended.

## Informed Consent

All participants obtained electronic informed consent for participation at the beginning of the questionnaire.

## Data Availability Statement

The raw data supporting the conclusions of this article will be made available by the authors, without undue reservation.

## Ethics Statement

The studies involving human participants were reviewed and approved by Institutional Review Board (IRB) Committees at Al-Balqa Applied University and the Hashimite University. The patients/participants provided their written informed consent to participate in this study.

## Author Contributions

TA-S, SS, KK, A-HA-M, SA-T, NA, and JA contributed to the conception and design of the study. TA-S organized the database and A-HA-M performed the statistical analysis. SA-T and AyA wrote the first draft of the manuscript. TA-S and A-HA-M interpreted the data for the work, wrote the final draft of the manuscript, and revised the manuscript critically for important intellectual content. AK, FA, LT, TS, WH, and NY contributed to data acquisition for the work. All authors contributed to the manuscript revision and approved the final version to be published. Agreement to be accountable for all aspects of the work in ensuring that questions related to the accuracy and integrity of the work parts were appropriately investigated and resolved.

## Conflict of Interest

The authors declare that the research was conducted in the absence of any commercial or financial relationships that could be construed as a potential conflict of interest.

## Publisher's Note

All claims expressed in this article are solely those of the authors and do not necessarily represent those of their affiliated organizations, or those of the publisher, the editors and the reviewers. Any product that may be evaluated in this article, or claim that may be made by its manufacturer, is not guaranteed or endorsed by the publisher.
